# Hemoglobin Response to a Low-Iron Dose in Infantile Anemia

**DOI:** 10.7759/cureus.107641

**Published:** 2026-04-24

**Authors:** Masahiko Kimura, Takeshi Taketani

**Affiliations:** 1 Pediatrics, Kimura Children and Family Clinic, Izumo, JPN; 2 Pediatrics, Shimane University Faculty of Medicine, Izumo, JPN

**Keywords:** gut microbiome, hemoglobin response, infant, iron-deficiency anemia, oxidative stress, therapeutic iron dose, therapeutic iron trial

## Abstract

Introduction: Iron-deficiency anemia (IDA) is common during infancy, especially among infants exclusively breastfed after six months of age. The recommended therapeutic iron dose is 3-6 mg/kg/day; however, excessive iron load can be harmful due to alteration of the gut microbiome and oxidative stress to the developing organs. Lower doses of iron have shown efficacy in treating IDA in adults. This retrospective study evaluated the hemoglobin (Hb) response to a lower iron dose in infantile anemia.

Methods: During health checkups for 9- to 10-month-old infants, anemia screening was conducted for those who were exclusively breastfed. Clinical parameters, including birth date, gestational age, birth weight, and body weight at the start of iron supplementation, were recorded. Venous blood samples were collected for a complete blood count (CBC). Infants with either Hb levels below 11.0 g/dL or mean corpuscular volume below 70 fL received a therapeutic iron trial. From March 2016 to November 2018, a fixed dose of elemental iron (soluble ferric pyrophosphate) 15 mg/day was administered (reference group), and from November 2018 to December 2023, a dose of 1 mg/kg/day was given (low-dose group). CBC measurements were repeated after four weeks, and Hb responses were compared between the two regimens.

Results: A total of 26 children in the reference group and 27 in the low-dose group were finally analyzed. The fixed 15 mg/day dose in the reference group corresponded to 1.8 (1.6-1.9) (median (interquartile range) mg/kg/day). Baseline clinical variables showed no statistically significant differences between the two groups. The median Hb increase was 1.6 g/dL and 1.5 g/dL, with 69% (18/26) and 70% (19/27) of children achieving an Hb level ≥ 1 g/dL, in the reference and low-dose groups, respectively. All 15 children (15/27, 56%) in the low-dose group, who met the new WHO anemia definition at the age of 6-23 months of Hb < 10.5 g/dL, had an Hb response ≥ 1.0 g/dL, with a median Hb response of 2.2 g/dL.

Conclusion: An iron dose of 1 mg/kg/day was as effective as 15 mg/day (median: 1.8 mg/kg/day) in the Hb response. In all children with Hb < 10.5 g/dL, the iron dose of 1 mg/kg/day showed substantial Hb responses. These findings suggest that therapeutic iron doses lower than conventional recommendations may be effective for managing IDA and mitigating the harmful effects.

## Introduction

Iron is essential not only for the synthesis of hemoglobin (Hb) but also for brain development [1]. Infantile iron-deficiency anemia (IDA) can occur at > 6 months of age in the absence of adequate exogenous iron sources. In Japan, where commercial iron-fortified foods and iron drops are generally unavailable, the prevalence of anemia in breastfed children has been reported to be as high as 40% [2].

Lower doses of iron have shown efficacy in treating IDA in the elderly [3] and pregnant women [4] and could reduce adverse effects, such as nausea and abdominal discomfort. However, in infants who are at risk for IDA, such studies of low iron supplementation have not been developed. Excessive iron can cause an unfavorable change in the gut microbiome [5], growth [6], and oxidative stress to developing organs in infants [7]. Furthermore, the regulation of iron absorption for iron overload is immature during infancy [8].

In a previous study conducted by our team, we identified exclusive breastfeeding as a risk factor for IDA in infancy [9]. In that study, children were treated with a fixed dose of elemental iron (soluble ferric pyrophosphate) 15 mg/day, equivalent to 1.5-2 mg/kg/day, and showed a good Hb response (unpublished data). Notably, this dose was lower than the conventional therapeutic range of 3-6 mg/kg/day [10]. Then, our team initiated the further use of a lower iron dose of 1 mg/kg/day for suspected IDA. The iron dose of 1 mg/kg/day was adopted because a randomized, placebo-controlled study of iron supplementation for prevention of exclusively breastfed infants showed some (as much as 34%) had an Hb response > 1.0 g/dL by the iron dose of 1 mg/kg/day (ferrous sulfate) [11,12]. The current study retrospectively evaluated the Hb response to a low iron dose. If a low dose of iron proves a good Hb response and can be applied to IDA, it would offer notable advantages, including ease in administration using iron drops and a lower likelihood of certain side effects that are often associated with higher iron doses, and prevent potential harmful effects of iron [7].

## Materials and methods

During routine health checkups for 9- to 10-month-old infants, screening for anemia was conducted for those who had been exclusively breastfed. Infants with either Hb levels below 11.0 g/dL or a mean corpuscular volume (MCV) below 70 fL were included in this study. We performed a therapeutic trial of iron for them as described by Oski [13] and Driggers et al. [14]. From March 2016 to November 2018, a group of infants received a fixed dose of elemental iron 15 mg/day (reference group), and from November 2018 to December 2023, another group of infants received a lower dose of elemental iron 1 mg/kg/day (low-dose group). The iron preparation was soluble ferric pyrophosphate (INCREMIN® syrup 5%; Alfresa Pharma Corporation, Osaka, Japan) [15]. According to the drug information about dosage and administration, children less than one year are given 2-4 mL (12-24 mg of the essential iron) per day, corresponding to 1.5-3 mg/kg /day for an 8-kg infant [15]. This was a liquid form of iron preparation only available to be prescribed for infants in Japan. Clinical parameters, including birth date, gestational age, birth weight, and body weight at the start of iron supplementation, were recorded. Following four weeks of iron supplementation, venous blood samples were collected to assess therapeutic responses using complete blood cell count analysis. This therapeutic trial of iron is a standard diagnostic approach for IDA, with an increase of ≥ 1.0 g/dL in Hb within one month [13,14].

The primary outcome was whether a dose of iron 1 mg/kg/day had a comparative Hb response to a dose of iron 15 mg/day, and a substantial Hb response compared to Hb responses to a conventional iron dose in the literature.

Laboratory Analysis

Venous blood samples were analyzed for Hb, MCV, and red cell distribution with coefficient variation (RDW-CV) using a blood counter (pocH-100i Sysmex; Kobe, Hyogo, Japan).

Statistical Analysis

For the analysis, we compared the clinical and laboratory data obtained for the low-dose and reference groups. In addition, all participants were stratified into two subgroups based on their Hb response, namely, those with an Hb increase ≥ 1.0 g/dL and those with an increase < 1.0 g/dL. Clinical variables were compared between the subgroups to identify potential predictors of Hb response. Hemoglobin cutoff to define anemia by WHO at the age of 6-23 months is < 10.5 g/dL [16]. According to this new definition, we analyzed the Hb responses of children with Hb < 10.5 g/dL in the low-dose and reference groups.

Descriptive statistical analyses were performed using Microsoft Excel (version 14; Microsoft Corporation, Redmond, WA). All data were presented as median and interquartile range (IQR) for skewness. Comparisons of clinical variables were conducted using Fisher's exact test for categorical data and the Mann-Whitney U test for continuous data. Differences were considered statistically significant at p < 0.05 (two-tailed). EZR (version 1.55; Saitama Medical Center, Jichi Medical University, Saitama, Japan) [17], a graphical user interface for R (version 4.0.3; The R Foundation for Statistical Computing, Vienna, Austria) was used.

Ethical Considerations

This study was approved by the Ethics Committee of Shimane University School of Medicine (Approval no. KS20240205-1) and performed with the principles of the Declaration of Helsinki. This study used an opt-out system, and the requirement for informed consent was waived.

## Results

**Figure 1:**
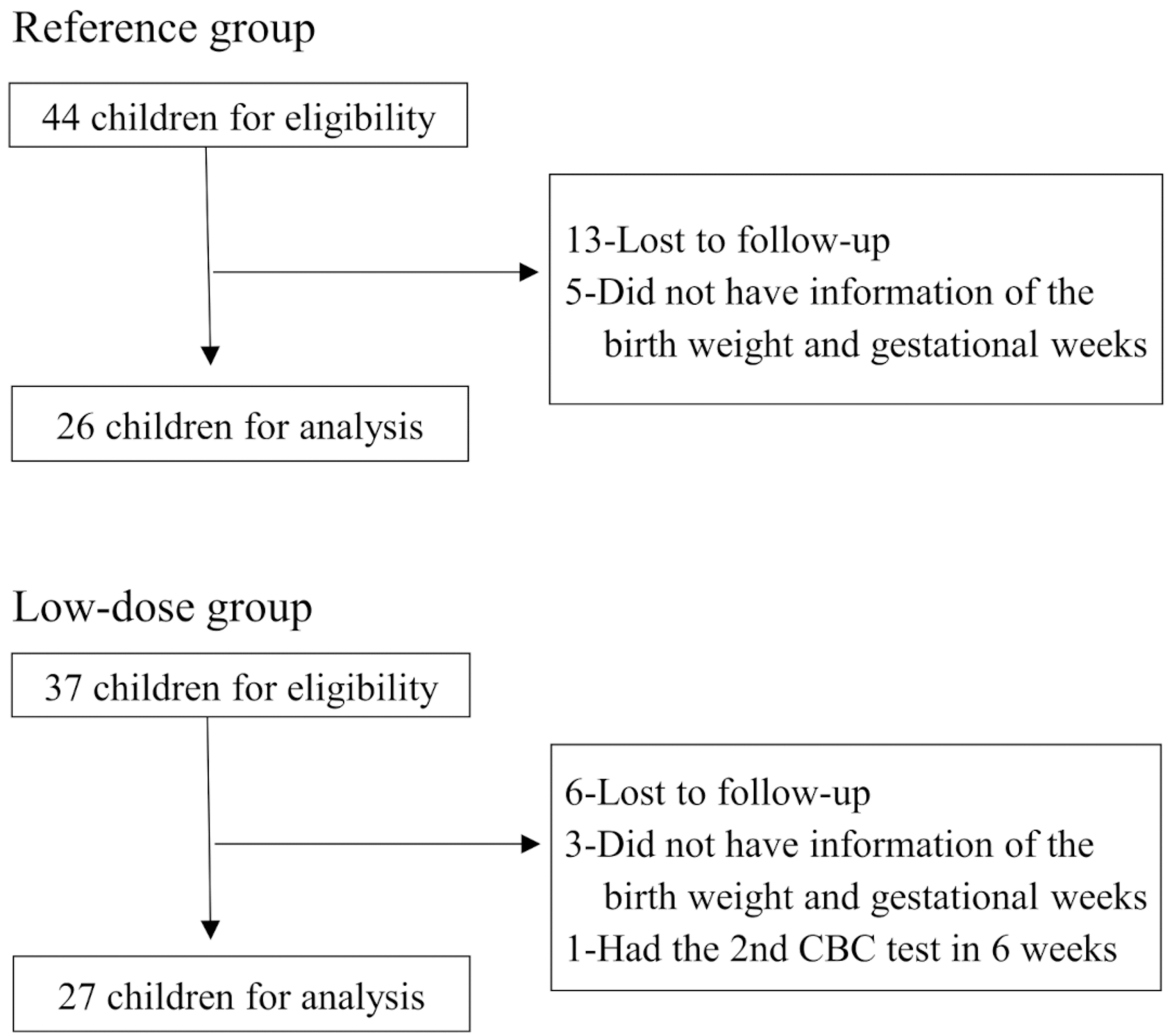
Flowchart of the reference and low-dose groups Eligible children were selected as (1) participants of 9- to 10-month-old routine health checkups; 2) exclusive breastfeeding; and 3) Hb levels below 11.0 g/dL or a mean corpuscular volume below 70 fL.

In the reference group in which children received a fixed iron dose of 15 mg /day from March 2016 to November 2018, 44 children were eligible for this study, and 26 of them were analyzed. The fixed dose of 15 mg/day was equivalent to 1.8 (1.7-1.9) mg/kg/day (median IQR). In the low-dose group in which children received an iron dose of 1 mg/kg/day from November 2018 to December 2023, 37 children were eligible for this study, and 27 of them were finally analyzed (Figure 1).

**Figure 2:**
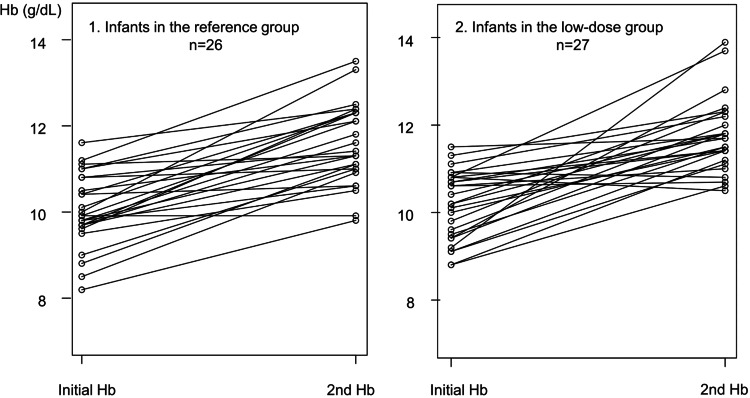
Hb changes before and after four weeks of the iron administration in the (1) reference and (2) low-dose groups

The baseline clinical variables were not different between the two groups. The median Hb increase was almost the same in both groups. The proportion of children achieving an Hb increase of ≥ 1.0 g/dL was 69% (18/26), 70% (19/27) in the reference and low-dose group, respectively (Table 1, Figure 2).

**Table 1:** Comparison of children receiving fixed iron dose of 15 mg/day, reference group) and low dose iron (1 mg/kg/day, low-dose group) IQR; Interquartile range, Δ: Amount of change, Hb: Hemoglobin; MCV: mean corpuscular volume, CV-RDW: coefficient of variation-red cell distribution width *Fisher exact test, **Mann-Whitney U test

Variables	Reference group	Low-dose group	p
Iron dose (mg/kg/day), median (IQR)	1.8 (1.6-1.9)	1.0	-
Number of children	26	27	-
Sex (male), n (%)	9 (35%)	18 (67%)	0.03*
Age at test (month), median (IQR)	9.5 (9-10)	9 (9-10)	-
Birth weight (g), median (IQR)	2906 (2569-3249)	2794 (2485-3096)	0.27**
Gestational age (day), median (IQR)	277 (267-281)	271 (267-277)	0.22**
Δ Weight (g), median (IQR)	5524 (5057-6122)	5397 (4913-5888)	0.53**
Initial Hb (g/dL), median (IQR)	9.9 (9.7-10.7)	10.2 (9.5-10.8)	0.66**
Initial MCV (fL), median (IQR)	66.9 (64.1-68.7)	65.5 (63.9-67.7)	0.30**
Initial CV-RDW (%), median (IQR)	16.5 (15.5-17.8)	16.3 (15.5-17.7)	0.74**
Δ Hb (g/dL), median (IQR)	1.6 (0.8-2.2)	1.5 (0.8-2.4)	0.98**
Δ MCV (fL), median (IQR)	2.9 (1.8-3.9)	4.2 (2.9-5.3)	0.12**
Δ CV-RDW (%), median (IQR)	4.1 (2.6-6.0)	4.0 (2.0-6.5)	0.94**
% Δ Hb ≥ 2.0	38% (10/26)	41% (11/27)	1*
% Δ Hb ≥ 1.0	69% (18/26)	70% (19/27)	1*
% Δ Hb ≥ 0.6	85% (22/26)	81% (22/27)	1*
Δ Hb in children with Δ Hb ≥ 1.0, median (range)	2.0 (1.5-2.3)	2.0 (1.4-2.4)	-

In total, 37 (70%) children had an Hb response of ≥ 1.0 g/dL (good responder), and 16 (30%) children had an Hb response < 1.0 g/dL (poor responder). When comparing the good and poor responders, the iron doses were similar between the groups. However, the good responders had significantly lower Hb, birth weights, and gestational ages than the poor responders (Table 2).

**Table 2:** Comparison of children with ≥ 1.0 g/dL and < 1.0 g/dL increment of the Hb response Δ: Amount of change, Hb: Hemoglobin, MCV: mean corpuscular volume, IQR: Interquartile range, CV-RDW: coefficient of variation-red cell distribution width *Fisher exact test, **Mann-Whitney U test

Variables	ΔHb ≥ 1.0 g/dL	ΔHb ＜ 1.0 g/dL	p
Number of children	37	16	-
Sex (male), n (%)	17 (46%)	10 (65%)	0.37*
Iron dose (mg/kg/day), median (IQR)	1.0 (1-1.8)	1.2 (1-1.7)	0.69**
Age at test (month), median (IQR)	9.0 (9-10)	9.5 (9-10.25)	0.19**
Birth weight (g), median (IQR)	2630 (2490-3000)	3098 (2888-3319)	0.005**
Gestational age (day), median (IQR)	273 (266-278)	279 (271-284)	0.045**
ΔWeight (g), median (IQR)	5488 (5057-5890)	5249 (4903-5962)	0.64**
Hb (g/dL) before therapy, median (IQR)	9.8 (9.4-10.2)	10.8 (9.8-10.9)	< 0.001**
MCV (fL) before therapy, median (IQR)	65.2 (61.9-67.4)	67.8 (66.2-68.9)	0.009**
CV-RDW (%) before therapy, median (IQR)	16.7 (15.8-18.5)	15.6 (15.3-16.5)	0.021**
ΔHb (g/dL), median (IQR)	2.0 (1.5-2.4)	0.3 (0.18-0.73)	-
ΔMCV (fL), median (IQR)	3.9 (2.9-5.4)	2.2 (0.95-3.6)	-
ΔCV-RDW (%), median (IQR)	5.3 (3.9-6.5)	1.9 (0.48-4.0)	-

**Figure 3:**
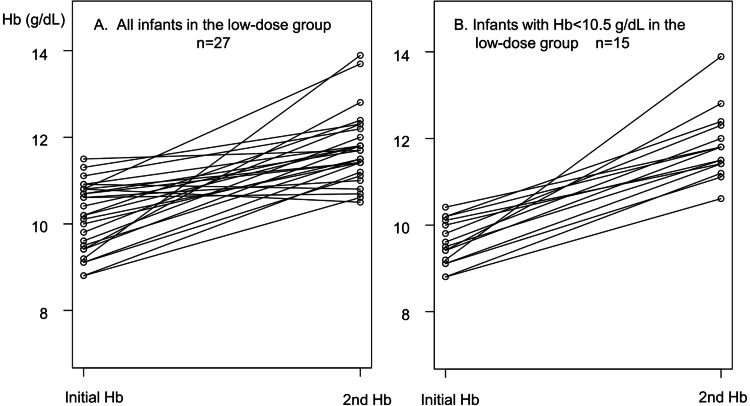
Hb changes before and after four weeks of the iron administration in all infants (A) and infants with the initial Hb < 10.5 g/dL (B) of the low-dose group

 Fifteen children (15/27, 56%) in the low-dose group and 18 (18/26, 69%) in the reference group had a WHO-defined anemia (Hb < 10.5 g/dL). All the children (15/15) in the low-dose group had an Hb response ≥ 1.0 g/dL, with a median Hb response of 2.2 g/dL, and 83% (15/18) in the reference group had an Hb response ≥ 1.0 g/dL, with a median Hb response of 2.0 g/dL (Table 3, Figure 3).

**Table 3:** Hb response in the reference and low-dose groups including only children with Hb < 10.5 g/dL Δ: amount of change, Hb: hemoglobin, IQR: interquartile range

Variables	Reference group	Low-dose group
Number of children	18	15
Δ Hb (g/dL), median (IQR)	2.0 (1.4-2.3)	2.2 (1.7-2.4)
Δ Hb ≥ 2.0	9 (50%)	8 (53%)
Δ Hb ≥ 1.0	15 (83%)	15 (100%)
Δ Hb ≥ 0.6	16 (89%)	15 (100%)

## Discussion

This study demonstrated that the reference and low-dose groups showed comparable hemoglobin (Hb) responses, with nearly 70% of children in both groups having an Hb response of ≥ 1.0 g/dL. The low-dose iron of 1 mg/kg/day showed a substantial Hb response of as much as 2.0 g/dL in four weeks. Although the optimal Hb response in infants is not known, an Hb response ≥ 1.0 g/dL within one month is thought to be reasonable [13,14,18], and even a ≥ 0.6 g/dL increase in Hb was a statistically significant response [14]. A study about the iron supplementation to exclusively breastfed infants suggested the Hb response of 0.5 or 1.0 g/dL to iron in infants aged six to nine months was a relevant criterion for IDA, and few children showed an Hb response of 1.5 or 2.0 g/dL [12].

A study with the conventional iron dose of 3.0 mg/kg/day using ferrous sulfate for three months in one-year-old infants with Hb level < 11.0 g/dL resulted in a 45% of response rate (Hb response ≥ 1.0 g/dL) and a mean Hb increase of 2.3 g/dL [14]. In contrast, our study found that 1.0 mg/kg/day of iron achieved a response rate of 70%, with a median Hb increase of 2.0 g/dL (Table 1) in the children with Hb response ≥ 1.0 g/dL. The higher response rate in our study, compared to the aforementioned study with the conventional iron doses [14], may reflect the iron-depleted state of exclusively breastfed infants in our study, regardless of the iron doses. They selected infants with Hb level <11.0 g/dL, not specifically exclusive breastfeeding [14]. Moreover, in our study, the good responders had lower birth weights and gestational ages, suggesting that they seemed to be in a further iron-depleted state in addition to exclusive breastfeeding. An iron-deficient state can promote iron absorption [19,20].

WHO in 2024 defined anemia at the Hb level < 10.5 g/dL in children aged 6-23 months [16]. Our study criteria were infants with either Hb levels below 11.0 g/dL or MCV below 70 fL. In our children with Hb < 10.5 g/dL, the Hb response rate and median Hb response value increased substantially in both groups. This means that children with an Hb level < 10.5 g/dL could be more iron-depleted, and the iron dose of 1 mg/kg was enough to give rise to a substantial Hb response.

A recent study evaluated the efficacy of ferric pyrophosphate (1 mg/kg/day), which was the same as our iron compound and dosage, but with a newer preparation of microencapsulation and liposomal formulation, compared to ferrous sulfate (3 mg/kg/day) [21]. It showed comparative efficacy of both regimens for pediatric IDA, namely, the efficacy of a low-iron dose. Regarding the difference in iron preparation, ferrous iron preparations have a generally good absorption, and, in particular, ferrous sulfate is widely used [19]. As mentioned earlier, ferrous sulfate of 1 mg/kg/day gave rise to an Hb response > 1.0 g/dL [11,12]. Our iron preparation has been constantly used for 60 years in Japan for IDA [15]. Although some new iron preparations have been developed for less gastrointestinal adverse effects [18,19], no one compound appears to be superior to another for hemoglobinization and repletion of iron stores [22].

An iron dose of 1.0 mg/kg/day, which showed substantial Hb responses in this study, was less than one-third of the conventional therapeutic iron dose and has been thought to be for the prevention of iron deficiency [23,24]. Notably, it closely approximates the estimated average daily iron requirement from 6-11 months (4.5 mg for boys and 4.0 mg for girls) [25]. Our study suggested that therapeutic iron doses lower than conventional recommendations may be effective for managing IDA. Although the adverse effects of iron supplementation for the growth and development of infants are inconclusive [7], a minimal iron dose for improving anemia needs to be defined.

This study had several limitations. First, this study was retrospective and conducted at a single clinic. Second, we evaluated the Hb response to iron for the therapeutic trial in infants with anemia. For the diagnosis of IDA, other biomarkers, such as serum ferritin, can be required. Third, factors such as the maternal history of anemia and intake of complementary food were not evaluated. Amano et al. reported that there was no correlation between infant and maternal Hb in exclusively breastfed infants [2]. In Japan, because commercial iron-fortified foods and iron drops are generally unavailable, iron sources from complementary food is limited, resulting in a high prevalence of anemia in breastfed children, which has been reported to be as high as 40% [2]. Fourth, the reference group received a fixed dose of 15 mg/day (median: 1.8 mg/kg/day), which was still lower than the conventional therapeutic dose. Fifth, we did not further explore non-responders who might be either physiological anemia or anemia of inflammation [22]. The new WHO anemia definition [16] can increase the specificity of the Hb response rate in our study.

Further prospective studies are needed to compare an Hb response to low and conventional iron doses, as well as adverse effects, and determine a minimum therapeutic iron dose. A desirable lower therapeutic iron dose can also make the preventive iron dose reconsidered. Furthermore, a study using ferrous sulfate, which is the most common iron preparation [18], would be applicable widely.

## Conclusions

This study demonstrated that a lower iron dose was sufficient for Hb response in iron-deplete infants. The low-iron dose of 1 mg/kg/day in this study has been considered as a preventive dose; however, it can be effective for treating infantile IDA, offering a viable alternative to the conventional iron therapy. Iron supplementation has been aimed at treating or preventing anemia without much attention to the harmful effects of iron. Further prospective studies of therapeutic iron doses are needed in infantile anemia.
